# Changes in Plasma Neutral and Ether-Linked Lipids Are Associated with The Pathology and Progression of Alzheimer’s Disease

**DOI:** 10.14336/AD.2023.0221

**Published:** 2023-10-01

**Authors:** Farida Dakterzada, Mariona Jové, Raquel Huerto, Anna Carnes, Joaquim Sol, Reinald Pamplona, Gerard Piñol-Ripoll

**Affiliations:** ^1^Unitat Trastorns Cognitius, Clinical Neuroscience Research, Hospital Universitari Santa Maria, IRBLleida, Lleida, Spain.; ^2^Department of Experimental Medicine, University of Lleida, IRBLleida, Lleida, Spain.; ^3^Institut Català de la Salut, Lleida, Spain.; ^4^Research Support Unit Lleida, Fundació Institut Universitari per a la Recerca a l'Atenció Primària de Salut Jordi Gol i Gurina (IDIAPJGol), Lleida, Spain.

**Keywords:** Alzheimer’s disease;, mild cognitive impairment, progression, lipidomics, plasma, ether-linked lipids

## Abstract

Aberrant lipid metabolism has been strongly linked to Alzheimer’s disease (AD) pathogenesis. However, the role of lipids in the pathophysiological processes of AD and their clinical progression is unclear. We hypothesized that plasma lipids are associated with the pathological hallmarks of AD, progression from mild cognitive impairment (MCI) to AD, and the rate of cognitive decline in MCI patients. To evaluate our hypotheses, we analysed the plasma lipidome profile by liquid chromatography coupled to mass spectrometry in an LC-ESI-QTOF-MS/MS platform for 213 subjects recruited consecutively: 104 AD, 89 MCI, and 20 control subjects. Forty-seven (52.8%) MCI patients progressed to AD during follow-up (58 ± 12.5 months). We found that higher plasma levels of sphingomyelin SM(36:0) and diglyceride DG(44:3) were associated with an increased risk of amyloid beta 42 (Aβ42) positivity in CSF, while levels of SM(40:1) were associated with a reduced risk. Higher plasma levels of ether-linked triglyceride TG(O-60:10) were negatively associated with pathological levels of phosphorylated tau in CSF. Plasma levels of fatty acid ester of hydroxy fatty acid FAHFA(34:0) and ether-linked phosphatidylcholine PC(O-36:1) were positively associated with pathological levels of total tau in CSF. Regarding the plasma lipids most associated with progression from MCI to AD, our analysis detected phosphatidyl-ethanolamine plasmalogen PE(P-36:4), TG(59:12), TG(46:0), and TG(O-62:7). Furthermore, TG(O-62:7) was the lipid that was most strongly associated with the rate of progression. In conclusion, our results indicate that neutral and ether-linked lipids are involved in the pathophysiological processes of AD and the progression from MCI to AD dementia, suggesting the involvement of lipid-mediated antioxidant mechanisms in AD.

## INTRODUCTION

Alzheimer’s disease (AD) is a human age-related pathological process that causes progressive neuro-degeneration. It is typically characterized by cognitive impairment and can ultimately affect behaviour, speech, visuospatial orientation, and the motor system. The increasing decline in these cognitive capacities affects a person’s ability to perform everyday activities and finally leads to a complete loss of independence, disability, and death [1]. AD accounts for 60-70% of all dementia cases. The global prevalence of dementia was estimated at 57.4 million cases in 2019. This number is predicted to increase to 152.8 million cases in 2050 [2].

From a pathological point of view, AD is currently characterized by the accumulation of extracellular abnormally folded amyloid-beta (Aβ) protein known as amyloid plaques, intracellular aggregations of hyperphosphorylated tau protein known as neurofibrillary tangles (NFTs), and neurodegeneration in the brain [3]. However, the exact aetiology of AD is still unknown, and it is believed that genetic, metabolic, bioenergetic, and environmental factors also have a role in the development of this disease [4,5].

The underlying pathophysiological processes of AD and its clinical symptomatology are best conceptualized as a continuum. Clinically, the AD continuum can be divided into three stages: preclinical, mild cognitive impairment (MCI), and AD dementia [6]. Neuro-degeneration, i.e., synaptic damage and neuronal death, is the pathological hallmark that has shown a good correlation with the decline in cognition [3]. However, the pathophysiological processes underlying neuro-degeneration are not completely understood. In addition, the rate of progression is highly variable among patients, with some having a faster course than others. The rate of progression from MCI to AD is the result of complex mechanisms interacting with each other. Heterogeneity in the rate of disease progression may be affected by disease and patient characteristics. Until now, research has mainly focused on discovering the risk factors for disease and the probability of developing AD regarding certain risk factors. However, our knowledge about the factors that may affect the disease trajectory is highly limited [7].

Lipids are an important class of biomolecules that are involved in many vital cellular processes, including their role as building blocks of the cell membrane, cell signalling, and energy storage [8]. Genetic [9] and clinical data [8] strongly link lipid dyshomeostasis to AD development. Consistent with this, the inheritance of the APOE ε4 allele is the strongest genetic risk factor for AD. APOE is involved in the transport and metabolism of fats, especially cholesterol [10]. In addition, many other genes related to lipid metabolism have also been identified as risk factors for AD [11]. Furthermore, some clinical conditions highly related to lipid dysregulation, such as obesity, cardiovascular diseases, and diabetes, are among the most frequent comorbidities of AD [12,13]. Many case-control studies have associated dysregulation in various classes of lipids with AD development [8]. However, little is known about the association between systemic lipid alterations and pathological hallmarks of the disease. In addition, there is a lack of longitudinal studies investigating the involvement of lipids in the pathophysiological processes of AD progression and those that may affect the course of cognitive evolution in this disease.

In this context, the objectives of the present study were as follows: first, to determine the association between plasma lipids and the clinical diagnosis of MCI and AD; second, to assess the relationship between plasma lipids and pathological hallmarks of the disease; third, to evaluate whether plasma lipids could be related to the MCI to AD progression; fourth, to identify the lipids that could be associated with the rate of cognitive decline in MCI patients; and fifth, to investigate whether alterations of lipid species in plasma could serve as prognostic biomarkers of progression and the rate of progression from MCI to AD.

## MATERIALS AND METHODS

### Study population

The study subjects were recruited consecutively from a sample of outpatients who visited the Cognitive Disorders Unit of the Hospital Universitari Santa Maria de Lleida from June 2014 to December 2016 and with the approval of the local ethics committee (CEIm 1374). The diagnosis of MCI and AD was performed according to the criteria of the National Institute on Ageing-Alzheimer’s Association [1,14]. Controls were recruited as subjects without neurological or neuropsychiatric diseases. Epidemiological data, including age, sex, and the time of symptom onset, were recorded using a structured interview conducted during the initial patient visit. Information regarding comorbidities and the levels of generic lipids, including total cholesterol, LDL cholesterol, HDL cholesterol, and TG, in serum was also obtained for each participant.

For AD and MCI patients, the exclusion criteria included a diagnosis of dementia other than AD or any somatic, psychiatric, or neurological disorder that might cause cognitive impairment or suffering from thyroid and/or vitamin B12 deficiency.

The MCI patients were followed up for a median of 58 (± 12.5) months to assess their progression to AD. The rate of progression from MCI to AD dementia was defined as the time between the baseline visit and the date of the diagnosis of AD. Progressive cognitive deterioration to AD was defined as (1) losing more than 3 points between the first and last Mini-Mental State Examination (MMSE), (2) having dementia at follow-up, or (3) getting a score less than 24 on the last MMSE [15].

### Cognitive evaluation

The cognitive state of the study population was assessed using the MMSE at baseline. For MCI patients, this test was applied at each annual visit until the end of the follow-up. The MMSE is a screening questionnaire for the detection of cognitive impairment. It contains 30 questions, with a maximum possible score of 30 points [16].

### Sample collection

Fasting blood and CSF samples were collected between 8:00 and 10:00 a.m. to avoid variations related to circadian rhythm. CSF samples were collected by a lumbar puncture at levels L4/L5 in polypropylene tubes. The samples were centrifuged at 2000 × g for 10 min at 4 °C to exclude cells or other insoluble material. The blood samples were collected in EDTA-containing tubes. The plasma was separated by centrifugation of blood samples at 1500 rpm for 20 min. The buffy coat was separated for DNA extraction and subsequent APOE genotyping. All samples were aliquoted and immediately stored at -80 °C until use. Samples were obtained with support from IRBLleida Biobank (B.0000682) and PLATAFORMA BIOBANCOS PT17/0015/0027, following the guidelines of Spanish legislation on this matter (Real Decreto 1716/2011).

### AD Biomarkers measurement

The levels of CSF Aβ42 (INNOTEST® β-AMYLOID (1-42)), total tau (Ttau) (INNOTEST® hTAU Ag), and phosphorylated tau (Ptau) (INNOTEST® PHOSPHO-TAU (181P)) were determined by ELISA based on the manufacturer’s instructions (Fujirebio Europe, Ghent, Belgium). We used our own cut-off points that were previously calculated based on another study population. Thus, we considered Aβ42 values < 600 pg/ml, Ttau > 425 pg/ml and Ptau > 65 pg/ml as positive/abnormal [17].

### APOE genotyping

DNA was extracted from the buffy coat cells automatically using Maxwell RSC Buffy Coat DNA Kit (Promega Biotech Ibérica SL, Madrid, Spain) on the Maxwell RSC instrument and according to the manufacturer’s instructions. APOE genotyping was performed by real-time PCR using 2.25 μl of extracted DNA and according to the TaqMan® SNP genotyping assay user guide (Publication Number MAN0009593, revision B.0).

### Lipidomics

The plasma lipidome was analysed using an untargeted lipidomic approach. The lipids were extracted using a methanol tert butyl ether-based validated method [18,19]. Class representative internal standards (Supplementary Table 1) were added to the extraction solvent to check lipid species retention time, to evaluate lipid extraction for each sample, and to use as internal standard for the semiquantitative approach used. Lipid extracts were analysed by liquid chromatography-mass spectrometry (LC-MS) using an Agilent UPLC 1290 liquid chromatograph coupled to an Agilent Q-TOF MS/MS 6520 mass spectrometer (Agilent Technologies, Barcelona, Spain) as previously described [20,21]. The order for the injection of samples was randomized, and quality control (QC) samples were used to control instrumental drif. Data were acquired in both positive and negative ionization modes. For MS/MS confirmation, the same parameters used for MS analyses were used, adding collision voltages of 0-V, 10-V, 20-V, and 40-V. Data were acquired using MassHunter Data Acquisition software (Agilent Technologies, Barcelona, Spain) and preprocessed using MassHunter Mass Profiler Professional software (Agilent Technologies, Barcelona, Spain), as previously described [21]. Only those features with a minimum of 2 ions were selected. Compounds from different samples were aligned using retention time windows of 0.1% ± 0.25 min and 30.0 ppm ± 2.0 mDa. Only stable features (found in at least 70% of the QC samples) were taken into account for the analysis and to correct for individual bias [22]. The signal was corrected using a LOESS approach [23]. QC samples were pools of all the samples distributed in different aliquots and inserted every five samples.

### Lipid identification

The differentially expressed features, defined by exact mass and retention time, were identified in the Human Metabolome Database (HMDB) [24], while the molecular weight tolerance was adjusted to 30 ppm. The adducts considered for the HMDB search are the following: positive ionization: M+H, M+NH4, M+NH4-H2O, M+Na, M+K, M+2K-H; negative ionization: M-H, M-H2O-H, M+C2H3O2, M+HCO2. Potential identities were confirmed by comparison of the exact mass and MS/MS spectra fragmentation pattern of the class representative internal standards, when available, with the public database [21], as well as comparing the retention time with the expected retention times of the chromatographic methodology used (Lysophospholipids: 0-3 min, Fatty acyls: 0-3.5 min, Phospholipids, Sphyngomyelins and Diacylglycerides: 3-7 min and Triacylglycerides and cholesteryl esters: 7.5-10.5 min) [20] and with the retention times of class-representative internal standards.

### Statistical analysis

One-way ANOVA (or nonparametric Kruskal-Wallis) and chi-square (or Fisher’s exact) tests were used for the analysis of the quantitative and qualitative variables among the three diagnostic groups, respectively. Student’s t (or the Mann-Whitney U) and chi-square (or Fisher’s exact) tests were used for the analysis of quantitative and qualitative variables between the progressive and nonprogressive groups. The quantitative variables are presented as the means (±standard deviation, SD) or medians (25th; 75th percentiles), and the qualitative variables are presented as percentages (frequency).

The cross-sectional association of experimental variables with quantitative outcomes (measured Aβ42, Ttau, and Ptau in the CSF and time to progression) was assessed using Spearman’s correlation. The cross-sectional association of the experimental variables with categorical outcomes (diagnosis, Aβ42 status, Ttau status, Ptau status, and progression/no progression) was studied using logistic regression models. Cox hazard analysis was used to assess the association of variables with the rate of progression. For all analyses, the values corresponding to each independent variable were dichotomized by their median, and the high value (> median) of each variable was compared to its low value (≤ median). A receiver operating curve (ROC) was provided to evaluate its quality. In addition, to determine whether the logistic regression models fit our data well, a Hosmer-Lemeshow test was performed for each model. The Hosmer-Lemeshow statistic indicates a poor fit if its significance value is less than 0.05. When analysing variables associated with progression, the AUC of the regression model, including lipids, was compared to the same model without lipids with the Hanley-McNeil test [25]. Values of z above the cut-off were taken as evidence that the “true” ROC areas are different. The adjustment of the level of significance to 1% (α = 0.01) and step-by-step forwards selection with conditional criteria were applied to minimize the negative effects of overfitting. The selection of variables by steps also allows the detection of multicollinearity. Detection of multicollinearity increases the precision of estimated coefficients and the power of the statistical analysis; however, it will also lose some variables that may be highly related to the dependent variables in the model (e.g., from the same metabolic pathway). To overcome this problem, after running each regression analysis, we eliminated the variables that had been input into the model, and the analysis was run again to let other influential lipids, if they exist, enter the model. In this way, we had several regression models for each comparison. This process was continued until the AUC of the model reached < 80. Finally, all of the statistical analyses were adjusted for age, sex, APOE ε4 allele, MMSE, and, if applicable, appropriate AD core CSF biomarkers (Aβ42, Ttau, and Ptau), including these parameters as predictors. All statistical analyses were performed using IBM SPSS version 25 (SPSS Inc., Chicago, IL, United States).

**Table 1:** Characteristics of the study population based on clinical diagnosis.

	Total (N = 213)	AD (N =104)	MCI (N = 89)	CTL (N = 20)	*p*
Demographic data					
Age	74 [70;78]	76 [72;80]	73 [69;78]	68 [62;74]	< 0.001
Sex (female)	54.5% (116)	58.6% (61)	50.6% (45)	50% (10)	0.530
Comorbidities					
Depression	31.4% (67)	27.8% (29)	40.9% (36)	10% (2)	0.014
Hypertension	53.2% (124)	58.6% (61)	62.5% (55)	40% (8)	0.183
Stroke	3.7% (8)	4.8% (5)	2.2% (2)	5% (1)	0.627
Diabetes Mellitus	19.7% (42)	18.2% (19)	21.6% (19)	20% (4)	0.847
Dyslipidemia	39.4% (84)	45.1% (47)	33% (29)	40% (8)	0.225
CSF AD biomarkers					
Aβ42 (pg/ml)	550 [419;711]	494 [395;583]	589 [435;864]	1016 [620;1347]	< 0.001
Ttau (pg/ml)	408 [252; 618]	489 [354;700]	329 [220;543]	248 [139;337]	< 0.001
Ptau (pg/ml)	67.8 [48;92]	81 [55;98]	62.7 [42;86]	45 [30;63]	< 0.001
MMSE	25 [23;27]	23.5 [22;25]	27 [25;28]	29.5 [28;30]	< 0.001
APOE ε4	44.6% (95)	52.9% (55)	42.7% (38)	10% (2)	0.002

AD: Alzheimer’s disease; MCI: mild cognitive impairment; CTL: control; MMSE: Mini-Mental State Examination; Aβ42: amyloid beta 1-42; Ttau: total tau; Ptau: phosphorylated tau; APOE ε4: apolipoprotein E ε4 allele; *P* values were calculated by comparing diagnostic groups using one-way ANOVA (or nonparametric Kruskal-Wallis tests) for quantitative variables and Chi-square tests for qualitative variables.

**Table 2:** Characteristics of progressive and nonprogressive MCI patients.

	Total MCI (N = 89)	Progressive MCI (N = 47)	Nonprogressive MCI (N = 42)	*p*
Demographic data				
Age	72 (6.0)	73 (6.0)	72 (5.1)	0.504
Sex (female)	51% (45)	53% (25)	48% (20)	0.600
Comorbidities				
Depression	40.4% (36)	40.4% (19)	40.4% (17)	0.996
Hypertension	62.9% (56)	55.3% (26)	71.4% (30)	0.116
Stroke	2.2% (2)	0% (0)	4.7% (2)	0.130
Diabetes Mellitus	21.3% (19)	19.1 (9)	23.8% (10)	0.592
Dyslipidemia	32.5% (29)	27.6% (13)	38.0% (16)	0.294
CSF AD biomarkers				
Aβ42 (pg/ml)	675[435;872]	481 [385;628]	805 [588;928]	< 0.001
Ttau (pg/ml)	413 [222;542]	482 [256;710]	265 [196;351]	< 0.001
Ptau (pg/ml)	76 [42;87]	76 [50;108]	54 [39;65]	< 0.001
MMSE	26 [25;28]	26 [24;28]	27 [26;29]	0.021
APOE ε4	44% (39)	67% (31)	19% (8)	< 0.001

MCI: mild cognitive impairment; Aβ42: amyloid beta 1-42; Ttau: total tau; Ptau: phosphorylated tau; MMSE: Mini-Mental State Examination; APOE ε4: apolipoprotein E ε4 allele;*P* values were calculated by comparing groups using Student’s t test (or Mann-Whitney U test) for quantitative variables and Pearson’s Chi-square test for qualitative variables

## RESULTS

### Study population

We included 213 participants that were divided into three diagnostic groups: 104 (46.8%) AD, 89 (41.5%) MCI, and 20 (9.4%) control (CTL) subjects (Table 1). The patients with MCI were followed up for a mean of 58 (± 12.5) months to evaluate their progression to AD. Our results showed that 47 patients (52.8%) progressed to AD, while 42 patients (47.2%) remained cognitively stable (Table 2). None of the MCI patients progressed to non-AD dementia.

### Plasma lipids associated with the diagnosis of MCI and AD

The plasma samples were analysed in positive and negative ionization modes. After baseline correction, peak picking and alignment, and further corrections, including quality control assessment, filtering, and correction of the signal, 1026 features remained for analysis, among which 607 molecules were detected in positive ionization mode and 419 in negative ionization mode.

Our analysis detected no lipids associated with the diagnosis of MCI and AD versus the control (Supplementary Fig. 1 and 2). However, when we conducted a logistic regression only between AD and MCI patients, we found 36 lipid species that were associated with the diagnosis. From these, we identified 13 lipid species that were mainly from the phospholipid class (Supplementary Table 2).

**Table 3:** Plasma lipids associated with the positivity of each AD-related CSF biomarker.

	Lipid name	Mass	Delta (ppm)	RT	IM	*p*	OR	99% CI for OR
Aβ42	SM(36:0)	770.5816	0	6.57	+	< 0.001	4.960	1.717-14.327
	SM(40:1)	786.6604	0	8.42	+	0.006	0.295	0.094-0.922
	DG(44:3)	730.6385	3	10.41	+	< 0.001	5.873	1.848-18.664
	Unknown	1338.195		10.46	+	0.009	0.289	0.084-0.990
Ptau	Unknown	1397.072		6.99	+	0.010	5.300	1.01-27.798
	TG(O-60:10)	978.7357	16	7.05	+	0.007	0.127	0.018-0.903
Ttau	FAHFA(34:0)	538.4952	13	3.65	-	0.004	3.106	1.122-8.600
	PC(O-36:1)	801.5872	8	7.39	-	0.009	2.613	1.015-7.704

Aβ42: amyloid beta 1-42; Ttau: total tau; Ptau: phosphorylated tau; RT: retention time (min); IM: ionization mode; OR: odds ratio. Delta = (abs (query mass - adduct mass)/adduct mass)*1000000MS

### Plasma lipids associated with CSF measures of AD pathology

To evaluate which lipid species could be associated with amyloid pathology, we divided the study population into two groups according to CSF Aβ42 levels: Aβ42 pathological (≤ 600 pg/mL) and no pathological (> 600 pg/mL). Our results showed that sphingomyelin SM (36:0) (*p*< 0.001), SM(40:1) (*p*< 0.001), diglyceride DG(44:3) (*p*< 0.001), and an unknown lipid (mass 1338.195, RT 10.46) (*p* = 0.009) were the most associated plasma lipids with Aβ42 positivity in CSF (Table 3). Regarding the association of plasma lipids with Ptau positivity in CSF, our analysis detected an ether-linked triglyceride TG(O-60:10) (*p*= 0.007) and an unknown lipid (mass 1397.072, RT 6.99) (*p* = 0.01) as the most associated lipids (Table 3). Finally, we found that fatty acid ester of hydroxy fatty acid FAHFA(34:0) (*p*= 0.004) and ether-linked phosphatidylcholine PC(O-36:1) (*p* = 0.009) were the most associated plasma lipids with pathological levels of Ttau in CSF (Table 3). Other lipids significantly associated with each AD-related pathology are presented in Supplementary Table 3. We did not detect any significant difference regarding comorbidities between subjects with and without pathological levels of CSF AD biomarkers (Supplementary Table 4, 5, and 6).

**Table 4:** Plasma lipids associated with progression from MCI to AD.

Lipid name	Mass	Delta (ppm)	RT	IM	*p*	OR	99% CI for OR
PE(P-36:4)	723.5188	3	7.25	+	0.003	0.005	0.000044-0.491
TG(59:12)	974.7156	25	7.4	+	0.002	98.654	1.989-4893.883
TG(46:0)	795.6105	5	7.87	+	0.002	64.863	2.050-2052.216
TG(O-62:7)	973.8682	26	10.27	+	0.002	0.013	0.000315-0.502

RT: retention time (min); IM: ionization mode; OR: odds ratio Delta = (abs(query mass - adduct mass)/adduct mass)*1000000MS

### Plasma lipids associated with progression from MCI to AD

The association of lipids with progression was evaluated by comparing the plasma lipidome profile of patients who progressed (N = 47) vs. patients who did not progress (N = 42) to AD during the follow-up. After adjusting for age, sex, APOE ε4, MMSE, and CSF levels of AD biomarkers, higher plasma levels of the two TGs were associated with a significantly greater risk of MCI to AD progression. These include TG(59:12) (*p*= 0.002) and TG(46:0) (*p*= 0.002). Higher plasma levels of phosphatidyl-ethanolamine plasmalogen PE(P-36:4) (*p*= 0.003) and TG(O-62:7) (*p*= 0.002) were associated with a significantly reduced risk of MCI to AD progression (Table 4). A regression model consisting of these four lipids and the APOE ε4 allele yielded an AUC = 0.972 (*p*< 0.001, 99% CI 0.936-1.000), and the Hosmer-Lemeshow test yielded a *p*= 0.957. To assess the effect of these lipids in discriminating progressive from nonprogressive MCI patients, we calculated the AUC of the model without these significantly associated lipids. Our results showed that the AUC of the model with only the APOE ε4 allele was 0.729 (*p* < 0.001, 99% CI 0.586-0.871). The results of the Hanley and McNeil tests indicated that the difference between the two AUCs was statistically significant (z = 4.595, |z| > 2.575, *p*< 0.01). Other lipids significantly associated with MCI to AD progression are shown in Supplementary Table 7.

**Table 5:** Plasma lipids associated with the rate of progression from MCI to AD

Name	Mass	Delta (ppm)	RT	IM	*p*	OR	99% CI for OR
APOE ε4					< 0.001	4.841	2.030-11.54
TG(O-62:7)	973.8682	26	10.27	+	< 0.001	0.307	0.135-0.696
Unknown	1038.717		9.06	-	0.004	2.461	1.107-5.471

RT: retention time (min); IM: ionization mode; OR: odds ratio Delta = (abs(query mass - adduct mass)/adduct mass)*1000000MS

### Plasma lipids associated with the rate of MCI to AD progression and the modelling rate of progression by use of associated plasma lipids

The association of lipids with the rate of progression (progression as a continuous dependent variable) was evaluated by the Cox hazard model. This regression model relates time to progression with the event of progression in the presence of influential variables.

**Figure 1.**
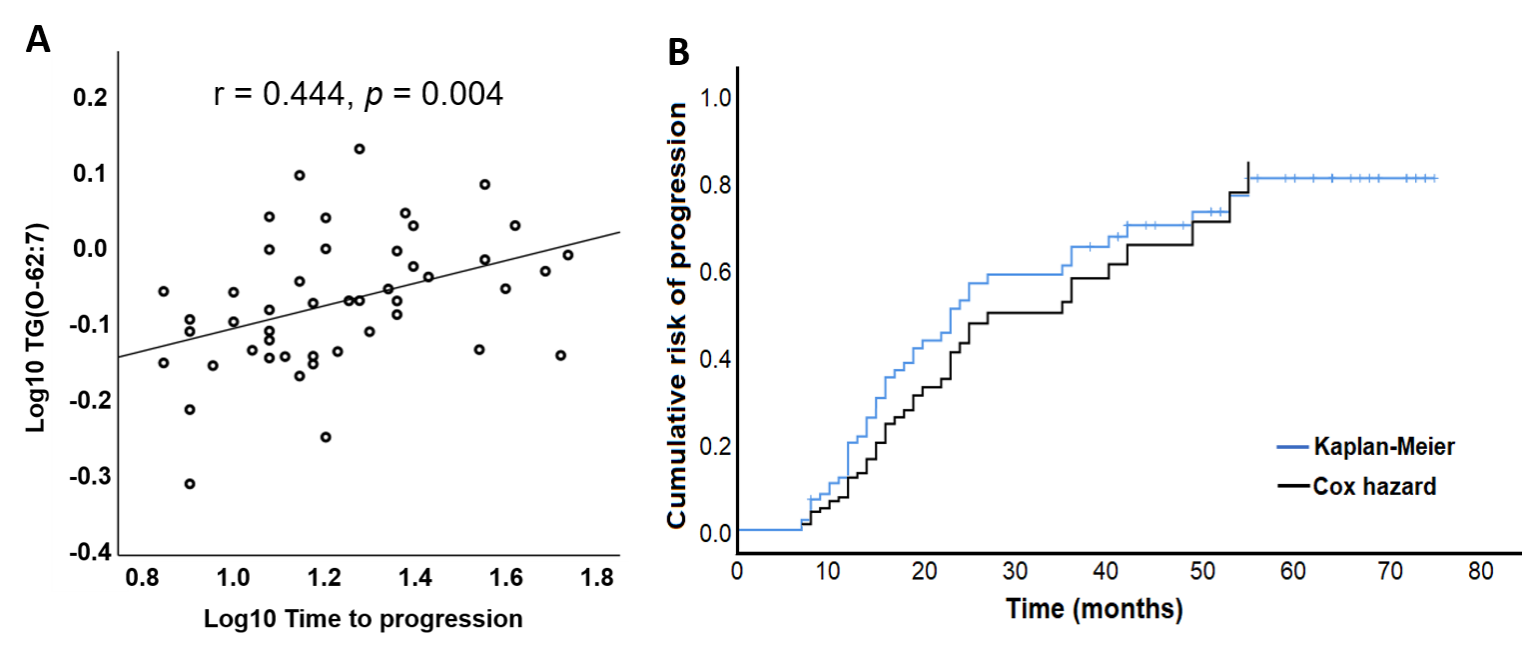
Association of lipids with the rate of MCI to AD progression. (A) TG(O-62:7) showed a significant positive correlation with the time to progression. (B) Comparison of the rate of progression predicted by the Cox hazard model (black line) with the real rate of progression of the MCI population calculated by Kaplan-Meier analysis (blue line).

Our analysis detected TG(O-62:7) and an unknown lipid (mass 1038.717, RT 9.06) as the most influential lipids in plasma associated with the rate of progression. Higher levels of TG(O-62:7) in plasma were associated with a lower rate of progression (*p*< 0.001), while higher levels of the unknown lipid were associated with a higher rate of progression (*p*= 0.004) (Table 5). In addition, TG(O-62:7) showed a highly positive correlation with time to progression (*r*= 0.444, *p*= 0.004) (Fig. 1A). Our data also confirmed the good accuracy of the Cox model in predicting the rate of MCI to AD progression compared to the real rate of progression calculated by Kaplan-Meier analysis (Fig. 1B). Other lipids significantly associated with the rate of MCI to AD progression are listed in Supplementary Table 8.

## DISCUSSION

In this study, we evaluated the relationships between plasma lipidomic data and both the clinical diagnosis of AD and the levels of AD biomarkers in CSF. We also searched for the association of lipids with MCI to AD progression and the rate of this progression. Our data were adjusted for multiple covariates, including age, sex, MMSE, APOE ε4, and AD core biomarkers. Controlling for these covariates was especially important to identify lipids specifically associated with each CSF biomarker, as well as to explore the involvement of lipids in MCI to AD progression independent of their possible role in the alterations of core AD biomarkers. We identified sets of lipids that were associated with each CSF biomarker and AD progression, suggesting that the role of lipids in AD pathology and progression is broader than their involvement in known pathological and cognitive aspects of AD and the influence of APOE ε4.

In the present study, we observed a dissociation between the current parameters of both MCI and AD diagnosis and the plasma lipidomic data since we did not find any lipid species associated with them. This lack of association might be a consequence of a divergent mechanistic relationship but also the result of the methodological constraints imposed in our analysis, which may have led to a lack of convergence in our statistical models. In fact, when we analysed only the data regarding the AD (N = 104) and MCI (N = 89) groups, we found several lipid species that were associated with the differential diagnosis between these two populations. In agreement with this lack of association between lipids and the differential diagnosis, Toledo et al. found no association between dysregulation of serum lipid species and the diagnosis of MCI and AD vs. control. These investigators were able to detect lipid dysregulations in participants with CSF pathology only after substratification of the diagnostic groups (control, MCI, and AD) based on CSF biomarkers [26].

We searched for specific associations of lipids with each AD CSF biomarker. We found two SMs and a DG that were associated with CSF Aβ42 status. The association of SM species has been related to CSF Aβ42 levels in previous reports [26,27]. Some studies have shown that Aβ42 can increase the production of DG via phospholipase D [8]. These associations all point to problems with membrane function and early neurodegeneration. In addition, SMs may be located in membrane lipid rafts, supporting the hypothesis that lipid rafts can directly regulate APP processing and Aβ42 production and facilitate its aggregation [28].

The association between ether-linked TGs and Ptau was not previously reported. Ether-linked TGs are present in LDs [29]. Therefore, the association between plasma levels of TG(O-60:10) and Ptau status in CSF suggests that LDs may have a role in tau pathology. LDs are dynamic and functionally active organelles involved in a variety of cellular functions, including energy and redox homeostasis [30]. Recently, it was shown that LDs can regulate proteasome activity and subsequently affect Ptau degradation [31]. In addition, considering the role of LDs in redox homeostasis, dysregulation regarding ether-linked TGs may implicate lipoxidation in Ptau pathology.

In our analysis, FAHFA(34:0) and PC(O-36:1) were associated with Ttau status in the CSF. Some ether-linked PC species were associated with Ttau measures in CSF [27] or the Ttau/Aβ42 ratio [26] in previous studies. FAHFAs are lipids consisting of a fatty acid coupled to a hydroxy fatty acid by an ester bond. It is assumed that the levels of FAHFAs in serum are mainly derived from endogenous synthesis. FAHFAs are mainly esterified to TGs and cholesteryl esters [32]. Therefore, these data again highlight the possible involvement of LDs in AD pathological processes. Some FAHFAs have been shown to have antioxidant properties by activating the transcription factor Nrf2 and increasing antioxidant enzymes [33]. Ether-linked PLs play a role as signalling molecules and they participate in the redox homeostasis of the membrane [34]. It is possible that these molecules, by modulating inflammation and oxidative stress, play an indirect role in the neurodegeneration process, or perhaps this dysregulation is an adaptive response to other pathological alterations in AD.

Some previous reports linked dysregulation regarding phospholipid and sphingolipid species to an increased risk of MCI to AD progression [26,27]. These studies did not take into consideration the measurement of neutral lipids. Our data suggest that neutral lipids may be involved in AD progression and the definition of the rate of progression. Neutral lipids are localized in LDs in cells [35]. Lipotoxicity is one of the principal causes of LD accumulation in the brain, especially in glial cells. Oxidized lipids produced in neurons are transported in a process mediated by APOE to surrounding astrocytes for their detoxification and storage in LDs [36]. Previous studies have found elevated lipoxidation markers in AD and MCI brains [37]. Therefore, lipoxidation could be a driving force behind neurodegeneration, and the protective mechanisms for reducing this neurotoxicity, such as LD formation, could affect the time to progression. Despite the efficiency of glia in sequestering peroxidated lipids, they have a limited capacity to do so. Glial cells eventually fail to resist the harmful consequences of peroxidated lipid storage, which finally leads to subsequent neuronal death [38]. On the other hand, dysregulation regarding TGs may not be a cause but rather a consequence of neuronal damage. However, LD-containing microglia have reduced phagocytic capacity, suggesting a possible feedback system where excessive LD accumulation in these cells impedes the rate of phagocytosis and finally leads to exacerbated neuronal loss [39].

At the systemic level, TGs are mainly produced in adipose tissue. The LDs in adipose tissue can affect physiological processes, such as inflammation [40]. Furthermore, LDs have been linked to peripheral metabolic dysfunction, such as insulin resistance and obesity [41], which are among the risk factors for AD. Therefore, peripheral neutral lipid dyshomeostasis may play a role in MCI to AD progression by increasing systemic inflammation and provoking general metabolic dysfunction that has been proven to affect the blood-brain barrier integrity and lead to increased AD pathology, synaptic damage and reduced cognition [42-44]. However, these neutral lipid dysregulations may not be the cause but rather the consequence of concomitant systemic metabolic abnormalities in AD patients.

Our finding regarding higher levels of PE(P-36:4) and a reduced risk of progression is in line with previous data that have linked the circulating levels of PE(P) to the cognitive performance of AD patients [45] and the risk of MCI to AD progression [26,27]. Considering that phospholipid plasmalogens are building blocks of cellular membranes and provide an optimal environment for the interaction, trafficking, and functions of proteins, dysregulation of these lipids may reasonably have detrimental effects on synapses and the clinical progression of AD. In a very recent study by Gu et al., intragastric administration of plasmalogens in an animal model of ageing promoted synaptic plasticity and neurogenesis, reduced neuroinflammation, and ameliorated the spatial learning and memory capacity of the animals [46]. These data indicate that not only central but also systemic homeostasis of these lipids could participate in the physiological processes underlying synaptic function and cognitive performance.

Furthermore, our results indicated that lipids significantly improve the prognostic efficacy of the existing markers of progression and, therefore, may have prognostic value in the detection of progressive MCI patients. In addition, measurement of systemic dysregulation of lipids may help in determining the rate of progression from MCI to AD.

Our study has some strengths and limitations. The strengths of our study include the following: first, we evaluated the association of lipids with each AD biomarker by controlling for other core AD biomarkers. This analysis permitted us to discover lipids that are specifically associated with each AD pathological aspect. Second, our MCI group had a long follow-up period that increased the accuracy of our defined groups as progressive or nonprogressive MCI. Third, for the first time, we assessed the association of lipids with AD diagnosis and progression, independent of their possible role in known pathological hallmarks of the disease. However, the small size of the control group was the major limitation of our study. In addition, some other confounders, such as medication and body mass index that were not taken into consideration in this study might have affected our results. Furthermore, our results should be confirmed in another cohort of patients and there is a need for studies, especially at the tissue level, to connect metabolic changes within a pathway and network context. In conclusion, our results indicate that neutral and ether-linked lipids are involved in the pathophysiological processes of AD pathology and the progression from MCI to AD dementia. The affected lipid species, based on their inherent functional properties, are linked to lipid-mediated antioxidant adaptive mechanisms at the cellular level, suggesting that lipid peroxidation plays role in AD pathology and progression. In addition, measurement of systemic dysregulation of lipids may help in determining the rate of progression from MCI to AD.

## Supplementary Materials

The Supplementary data can be found online at: www.aginganddisease.org/EN/10.14336/AD.2023.0220.



## Data Availability

The metabolomics dataset used for the analyses is available in https://doi.org/10.34810/data614.

## References

[1] McKhann GM, Knopman DS, Chertkow H, Hyman BT, Jack CR, Kawas CH, et al (2011). The diagnosis of dementia due to Alzheimer’s disease: Recommendations from the National Institute on Aging-Alzheimer’s Association workgroups on diagnostic guidelines for Alzheimer’s disease. Alzheimers Dement, 7: 263-69. 21514250 10.1016/j.jalz.2011.03.005 PMC3312024

[2] Nichols E, Steinmetz JD, Vollset SE, Fukutaki K, Chalek J, Abd-Allah F, et al (2022). Estimation of the global prevalence of dementia in 2019 and forecasted prevalence in 2050: an analysis for the Global Burden of Disease Study 2019. Lancet Public Heal, 7: 105-25. 10.1016/S2468-2667(21)00249-8 PMC8810394 34998485

[3] Jack CR, Bennett DA, Blennow K, Carrillo MC, Dunn B, Haeberlein SB, et al (2018). NIA-AA Research Framework: Toward a biological definition of Alzheimer’s disease. Alzheimers Dement, 14:535-62. 29653606 10.1016/j.jalz.2018.02.018 PMC5958625

[4] Jové M, Mota-Martorell N, Torres P, Ayala V, Portero-Otin M, Ferrer I, et al (2021). The Causal Role of Lipoxidative Damage in Mitochondrial Bioenergetic Dysfunction Linked to Alzheimer’s Disease Pathology. Life, 11:388. 33923074 10.3390/life11050388 PMC8147054

[5] Ferrer I (2022). Hypothesis review: Alzheimer’s overture guidelines. Brain Pathol, e13122. 36223647 10.1111/bpa.13122 PMC9836379

[6] Dubois B, Hampel H, Feldman HH, Scheltens P, Aisen P, Andrieu S, et al (2016). Preclinical Alzheimer’s disease: Definition, natural history, and diagnostic criteria. Alzheimers Dement, 12:292. 27012484 10.1016/j.jalz.2016.02.002 PMC6417794

[7] Melis RJF, Haaksma ML, Muniz-Terrera G (2019). Understanding and predicting the longitudinal course of dementia. Curr Opin Psychiatry, 32:123. 30557268 10.1097/YCO.0000000000000482 PMC6380437

[8] Kao YC, Ho PC, Tu YK, Jou IM, Tsai KJ (2020). Lipids and Alzheimer’s Disease. Int J Mol Sci, 21:1505. 32098382 10.3390/ijms21041505 PMC7073164

[9] Kunkle BW, Grenier-Boley B, Sims R, Bis JC, Damotte V, Naj AC, et al (2019). Genetic meta-analysis of diagnosed Alzheimer’s diseaseidentifies new risk loci and implicates Aβ, tau, immunity and lipidprocessing. Nat Genet, 51:414. 30820047 10.1038/s41588-019-0358-2 PMC6463297

[10] Huynh T-P V, Davis AA, Ulrich JD, Holtzman DM (2017). Apolipoprotein E and Alzheimer’s disease: the influence of apolipoprotein E on amyloid-β and other amyloidogenic proteins. J Lipid Res, 58:824-36. 28246336 10.1194/jlr.R075481 PMC5408619

[11] Bellenguez C, Grenier-Boley B, Lambert JC (2020). Genetics of Alzheimer’s disease: where we are, and where we are going. Curr Opin Neurobiol, 61:40-8. 31863938 10.1016/j.conb.2019.11.024

[12] Edwards GA, Gamez N, Escobedo G, Calderon O, Moreno-Gonzalez I (2019). Modifiable risk factors for Alzheimer’s disease. Front Aging Neurosci, 11:146. 31293412 10.3389/fnagi.2019.00146 PMC6601685

[13] Armstrong RA, Richard P, Armstrong A (2019). Risk factors for Alzheimer’s disease. Folia Neuropathol, 57:87-105. 31556570 10.5114/fn.2019.85929

[14] Albert MS, DeKosky ST, Dickson D, Dubois B, Feldman HH, Fox NC, et al (2011). The diagnosis of mild cognitive impairment due to Alzheimer’s disease: recommendations from the National Institute on Aging-Alzheimer’s Association workgroups on diagnostic guidelines for Alzheimer’s disease. Alzheimers Dement, 7:270-9. 21514249 10.1016/j.jalz.2011.03.008 PMC3312027

[15] Caroli A, Prestia A, Galluzzi S, Ferrari C, Van Der Flier WM, Ossenkoppele R, et al (2015). Mild cognitive impairment with suspected nonamyloid pathology (SNAP): Prediction of progression. Neurology, 84:508-15. 25568301 10.1212/WNL.0000000000001209 PMC4336071

[16] Folstein MF, Folstein SE, McHugh PR (1975). “Mini-mental state”: A practical method for grading the cognitive state of patients for the clinician. J Psychiatr Res, 12:189-98. 1202204 10.1016/0022-3956(75)90026-6

[17] Ortega RL, Dakterzada F, Arias A, Blasco E, Naudí A, Garcia FP, et al (2019). Usefulness of CSF Biomarkers in Predicting the Progression of Amnesic and Nonamnesic Mild Cognitive Impairment to Alzheimer’s Disease. Curr Aging Sci, 12:35-42. 31589110 10.2174/1874609812666190112095430

[18] Pizarro C, Arenzana-Rámila I, Pérez-Del-Notario N, Pérez-Matute P, González-Sáiz JM (2013). Plasma lipidomic profiling method based on ultrasound extraction and liquid chromatography mass spectrometry. Anal Chem, 85:12085-92. 24266777 10.1021/ac403181c

[19] Sol J, Jové M, Povedano M, Sproviero W, Domínguez R, Piñol-Ripoll G, et al (2021). Lipidomic traits of plasma and cerebrospinal fluid in amyotrophic lateral sclerosis correlate with disease progression. Brain Commun, 3:fcab143. 34396104 10.1093/braincomms/fcab143 PMC8361390

[20] Castro-Perez JM, Kamphorst J, Degroot J, Lafeber F, Goshawk J, Yu K, et al (2010). Comprehensive LC-MS E lipidomic analysis using a shotgun approach and its application to biomarker detection and identification in osteoarthritis patients. J Proteome Res, 9:2377-89. 20355720 10.1021/pr901094j

[21] Jové M, Cabré R, Mota-Martorell N, Martin-Garí M, Obis È, Ramos P, et al (2021). Age-related changes in lipidome of rat frontal cortex and cerebellum are partially reversed by methionine restriction applied in old age. Int J Mol Sci, 22:22. 10.3390/ijms222212517 PMC8623997 34830402

[22] Broadhurst D, Goodacre R, Reinke SN, Kuligowski J, Wilson ID, Lewis MR, et al (2018). Guidelines and considerations for the use of system suitability and quality control samples in mass spectrometry assays applied in untargeted clinical metabolomic studies. Metabolomics. 14:72. 29805336 10.1007/s11306-018-1367-3 PMC5960010

[23] Dunn WB, Broadhurst D, Begley P, Zelena E, Francis-Mcintyre S, Anderson N, et al (2011). Procedures for large-scale metabolic profiling of serum and plasma using gas chromatography and liquid chromatography coupled to mass spectrometry. Nat Protoc, 6:1060-83. 21720319 10.1038/nprot.2011.335

[24] Wishart DS, Feunang YD, Marcu A, Guo AC, Liang K, Vázquez-Fresno R, et al (2018). HMDB 4.0: the human metabolome database for 2018. Nucleic Acids Res, 46:D608-17. 29140435 10.1093/nar/gkx1089 PMC5753273

[25] Hanley JA, McNeil BJ (1983). A method of comparing the areas under receiver operating characteristic curves derived from the same cases. Radiology, 148:839-43. 6878708 10.1148/radiology.148.3.6878708

[26] Toledo JB, Arnold M, Kastenmüller G, Chang R, Baillie RA, Han X, et al (2017). Metabolic network failures in Alzheimer’s disease: A biochemical road map. Alzheimer Dement 13:965-84. 10.1016/j.jalz.2017.01.020 PMC5866045 28341160

[27] Varma VR, Oommen AM, Varma S, Casanova R, An Y, Andrews RM, et al (2018). Brain and blood metabolite signatures of pathology and progression in Alzheimer disease: A targeted metabolomics study. PLoS Med, 15: e1002482. 29370177 10.1371/journal.pmed.1002482 PMC5784884

[28] Di Paolo G, Kim TW (2011). Linking lipids to Alzheimer’s disease: cholesterol and beyond. Nat Rev Neurosci, 12:284-96. 21448224 10.1038/nrn3012 PMC3321383

[29] Gandotra S (2020). Lipid droplets in the immune response and beyond. Lipid Signal Metab, 1:173-96.

[30] Farmer BC, Walsh AE, Kluemper JC, Johnson LA (2020). Lipid Droplets in Neurodegenerative Disorders. Front Neurosci, 14:742. 32848541 10.3389/fnins.2020.00742 PMC7403481

[31] van der Kant R, Langness VF, Herrera CM, Williams DA, Fong LK, Leestemaker Y, et al (2019). Cholesterol Metabolism Is a Druggable Axis that Independently Regulates Tau and Amyloid-β in iPSC-Derived Alzheimer’s Disease Neurons. Cell Stem Cell 24:363-75. 30686764 10.1016/j.stem.2018.12.013 PMC6414424

[32] Riecan M, Paluchova V, Lopes M, Brejchova K, Kuda O (2022). Branched and linear fatty acid esters of hydroxy fatty acids (FAHFA) relevant to human health. Pharmacol Ther, 231:107972. 34453998 10.1016/j.pharmthera.2021.107972

[33] Gowda SGB, Fuda H, Tsukui T, Chiba H, Hui SP (2020). Discovery of Eicosapentaenoic Acid Esters of Hydroxy Fatty Acids as Potent Nrf2 Activators. Antioxidants (Basel), 9:397. 32397146 10.3390/antiox9050397 PMC7278747

[34] Dean JM, Lodhi IJ (2018). Structural and functional roles of ether lipids. Protein Cell, 9:196. 28523433 10.1007/s13238-017-0423-5 PMC5818364

[35] Olzmann JA, Carvalho P (2018). Dynamics and functions of lipid droplets. Nat Rev Mol Cell Biol, 20:137-55. 10.1038/s41580-018-0085-z PMC6746329 30523332

[36] Ioannou MS, Jackson J, Sheu SH, Chang CL, Weigel A V., Liu H, et al (2019). Neuron-Astrocyte Metabolic Coupling Protects against Activity-Induced Fatty Acid Toxicity. Cell, 177:1522-35. 31130380 10.1016/j.cell.2019.04.001

[37] Zabel M, Nackenoff A, Kirsch WM, Harrison FE, Perry G, Schrag M (2018). Markers of oxidative damage to lipids, nucleic acids and proteins and antioxidant enzymes activities in Alzheimer’s disease brain: A meta-analysis in human pathological specimens. Free Radic Biol Med, 115:351-60. 29253591 10.1016/j.freeradbiomed.2017.12.016 PMC6435270

[38] Moulton MJ, Barish S, Ralhan I, Chang J, Goodman LD, Harland JG, et al (2021). Neuronal ROS-induced glial lipid droplet formation is altered by loss of Alzheimer’s disease-associated genes. Proc Natl Acad Sci U S A, 118: e2112095118. 34949639 10.1073/pnas.2112095118 PMC8719885

[39] Ralhan I, Chang CL, Lippincott-Schwartz J, Ioannou MS (2021). Lipid droplets in the nervous system. J Cell Biol, 220: e202102136. 34152362 10.1083/jcb.202102136 PMC8222944

[40] Jarc E, Petan T (2020). A twist of FATe: Lipid droplets and inflammatory lipid mediators. Biochimie, 169:69-87. 31786231 10.1016/j.biochi.2019.11.016

[41] Konige M, Wang H, Sztalryd C (2014). Role of adipose specific lipid droplet proteins in maintaining whole body energy homeostasis. Biochim Biophys Acta - Mol Basis Dis, 1842:393-401. 10.1016/j.bbadis.2013.05.007 PMC4507416 23688782

[42] Kinney JW, Bemiller SM, Murtishaw AS, Leisgang AM, Salazar AM, Lamb BT (2018). Inflammation as a central mechanism in Alzheimer’s disease. Alzheimer’s Dement Transl Res Clin Interv, 4:575. 10.1016/j.trci.2018.06.014 PMC6214864 30406177

[43] Maiuolo J, Gliozzi M, Musolino V, Carresi C, Scarano F, Nucera S, et al (2021). From Metabolic Syndrome to Neurological Diseases: Role of Autophagy. Front Cell Dev Biol, 9:619. 10.3389/fcell.2021.651021 PMC8017166 33816502

[44] Sankowski R, Mader S, Valdés-Ferrer SI (2015). Systemic Inflammation and the Brain: Novel Roles of Genetic, Molecular, and Environmental Cues as Drivers of Neurodegeneration. Front Cell Neurosci, 9:28. 25698933 10.3389/fncel.2015.00028 PMC4313590

[45] Wood PL, Mankidy R, Ritchie S, Heath D, Wood JA, Flax J, et al (2010). Circulating plasmalogen levels and Alzheimer Disease Assessment Scale-Cognitive scores in Alzheimer patients. J Psychiatry Neurosci, 35:59. 20040248 10.1503/jpn.090059 PMC2799506

[46] Gu J, Chen L, Sun R, Wang JL, Wang J, Lin Y, et al (2022). Plasmalogens Eliminate Aging-Associated Synaptic Defects and Microglia-Mediated Neuroinflammation in Mice. Front Mol Biosci, 9:159. 10.3389/fmolb.2022.815320 PMC8906368 35281262

