# Supplementary material for: Changes in Plasma Neutral and Ether-Linked Lipids Are Associated with The Pathology and Progression of Alzheimer’s Disease
Source: Aging Dis. 2023 Oct 1;14(5):1728–38. doi: 10.14336/AD.2023.0221 (PMC10529749; doi:10.14336/AD.2023.0221)
Supplement: Supplementary file 1 [file AD-14-5-1728-s.pdf]

SUPPLEMENTARY DATA

Changes in Plasma Neutral and Ether-Linked Lipids Are Associated with The Pathology and Progression of Alzheimer's Disease

Farida Dakterzada, Mariona Jové, Raquel Huerto, Anna Carnes, Joaquim Sol, Reinald Pamplona, Gerard Piñol-Ripoll

© 2023. Dakterzada F et al. Published online at http://www.aginganddisease.org/EN/10.14336/AD.2023.0220

SUPPLEMENTARY DATA

Supplementary Table 1. Class representative and extraction internal standards added to the samples.

**Compound**	**Reference (Catalogue number, provider)**
1,3(d5)-dihexadecanoyl-glycerol	110537, Avanti Polar Lipids
1,3(d5)-dihexadecanoyl-2-octadecanoyl-glycerol	110543, Avanti Polar Lipids
1-hexadecanoyl(d31)-2-(9Z-octadecenoyl)-sn-glycero-3-phosphate	110920, Avanti Polar Lipids
1-hexadecanoyl(d31)-2-(9Z-octadecenoyl)-sn-glycero-3-phosphocholine	110918, Avanti Polar Lipids
1-hexadecanoyl(d31)-2-(9Z-octadecenoyl)-sn-glycero-3-phosphoethanolamine	110921, Avanti Polar Lipids
1-hexadecanoyl-2-(9Z-octadecenoyl)-sn-glycero-3-phospho-(1'-rac-glycerol-1',1',2',3',3'-d5)	110899, Avanti Polar Lipids
1-hexadecanoyl(d31)-2-(9Z-octadecenoyl)-sn-glycero-3-phospho-myo-inositol	110923, Avanti Polar Lipids
1-hexadecanoyl(d31)-2-(9Z-octadecenoyl)-sn-glycero-3-[phospho-L-serine]	110922, Avanti Polar Lipids
26:0-d4 Lyso PC	860389, Avanti Polar Lipids
18:1 Chol (D7) ester	111015, Avanti Polar Lipids
cholest-5-en-3ß-ol(d7)	LM-4100, Avanti Polar Lipids
D-erythro-sphingosine-d7	860657, Avanti Polar Lipids
D-erythro-sphingosine-d7-1-phosphate	860659, Avanti Polar Lipids
N-palmitoyl-d31-D-erythro-sphingosine	868516, Avanti Polar Lipids
N-palmitoyl-d31-D-erythro-sphingosylphosphorylcholine	868584, Avanti Polar Lipids
Octadecanoic acid-2,2-d2	19905-58-9, Sigma Aldrich

© 2023. Dakterzada F et al. Published online at http://www.aginganddisease.org/EN/10.14336/AD.2023.0220

SUPPLEMENTARY DATA

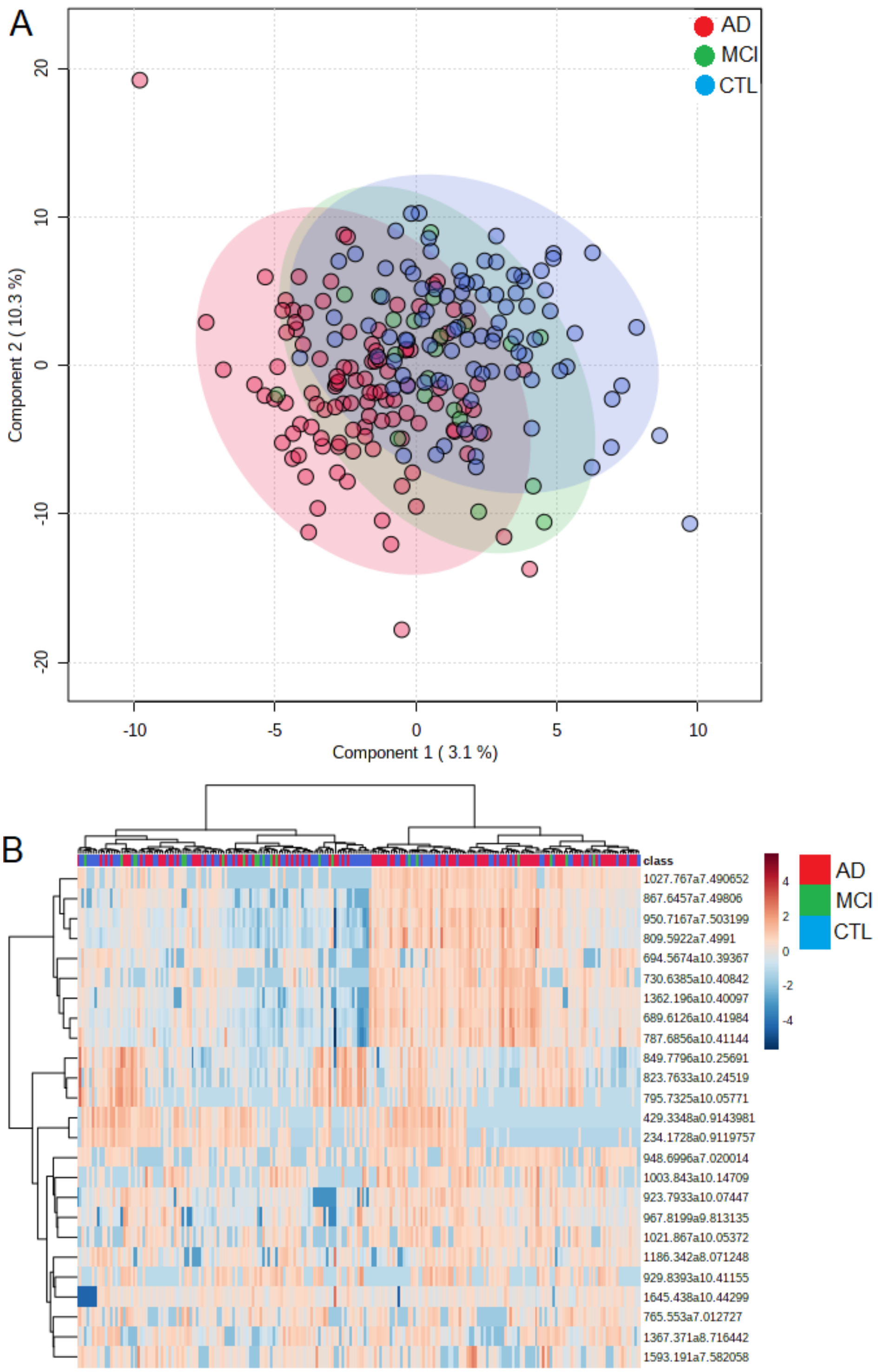

Supplementary Figure 1. Plasma lipidomic profile of patients with AD, MCI, and control subjects detected in positive ionization mode. (A) Partial least squares-discriminant analysis for the diagnostic groups. (B) The heat map representation of the top 25 lipids with significantly different (not FDR adjusted) levels between three diagnostic groups.

© 2023. Dakterzada F et al. Published online at http://www.aginganddisease.org/EN/10.14336/AD.2023.0220

SUPPLEMENTARY DATA

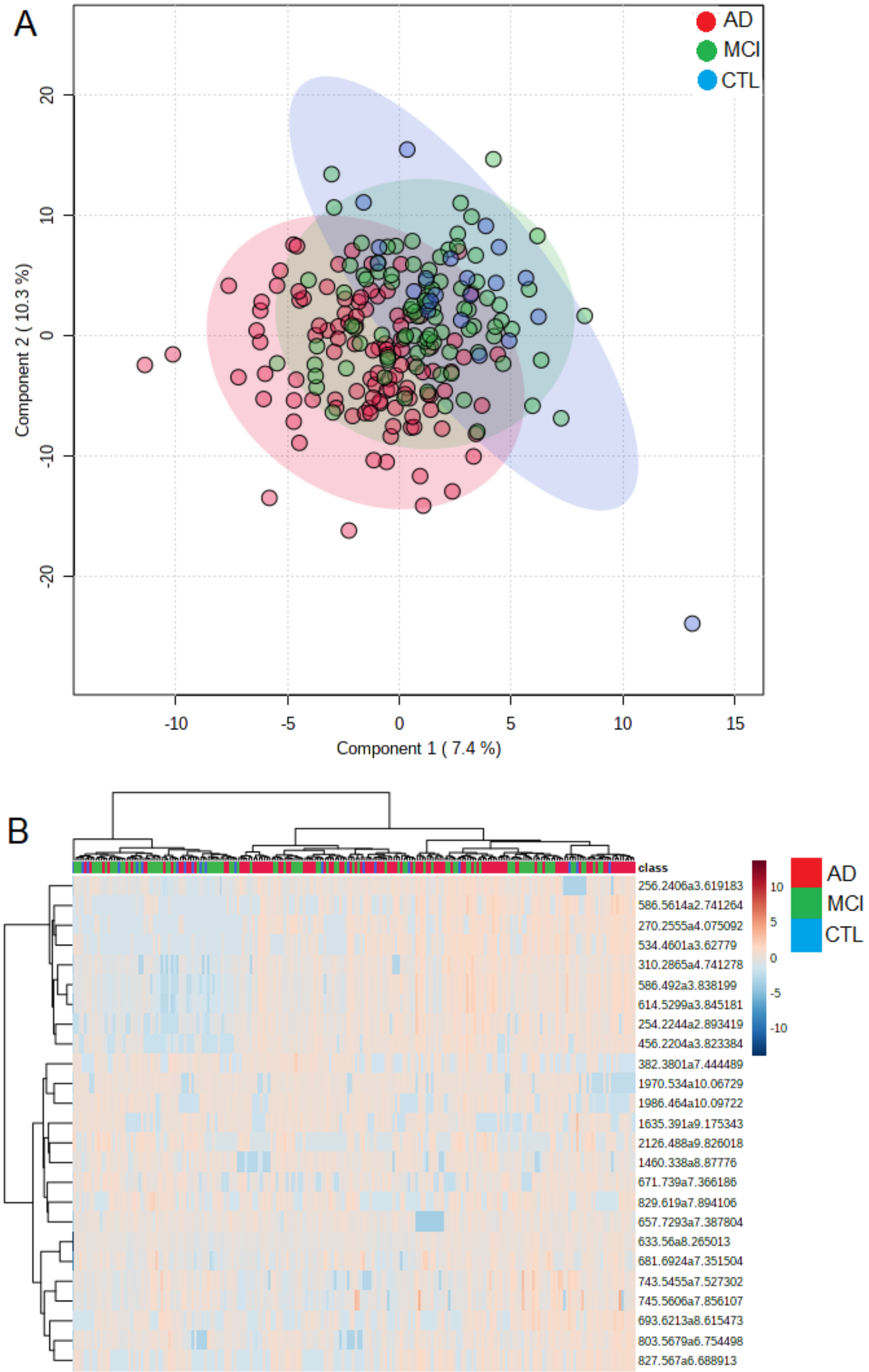

Supplementary Figure 2. Plasma lipidomic profile of patients with AD, MCI, and control subjects detected in negative ionization mode. (A) Partial least squares-discriminant analysis of diagnostic groups. (B) The heat map representation of the top 25 lipids with significantly different (not FDR adjusted) levels between three diagnostic groups.

© 2023. Dakterzada F et al. Published online at http://www.aginganddisease.org/EN/10.14336/AD.2023.0220

SUPPLEMENTARY DATA

Supplementary Table 2. Plasma lipids associated with diagnosis (AD vs MCI). MCI was set as the reference category.

Name	Mass	Delta (ppm)	RT	*p*	IM	OR	99% CI for OR
PC(38:5)	807.5754	13	7.01	0.008	+	4.794	1.055 - 21.789
Unknown	727.221		8.36	0.005	+	4.332	1.136 - 16.522
HexCer(d18:1/12:0)	642.5185	25	8.36	< 0.001	+	20.24	3.503 - 116.93
PC(O-44:6)	875.6767	9	8.42	< 0.001	+	28.58	4.954 - 164.92
Unknown	194.2805		10.69	< 0.001	+	0.095	0.017 - 0.522
Unknown	192.1746		10.74	< 0.001	+	0.063	0.011 - 0.356
nknown	429.3348		0.91	< 0.001	+	0.115	0.028 - 0.481
TG(56:3)	929.8393	10	10.41	0.002	+	0.202	0.052 - 0.781
PC(2OH-46:6)	948.6996	24	7.02	0.002	+	3.273	1.209 - 8.860
Unknown	146.5434		8.72	0.008	+	2.8	1.03 - 7.614
Unknown	234.1728		0.92	0.004	+	0.327	0.119 - 0.897
PC(40:7)	831.5754	12	6.9	0.003	+	3.385	1.177 - 9.735
Unknown	833.2103		7.62	0.005	+	3.051	1.087 - 8.565
CE(15D5)	774.7006	5	10.52	< 0.001	+	0.198	0.064 - 0.614
Unknown	466.3116		0.91	0.003	−	0.220	0.058 - 0.833
C20:1 (n-7)	310.2865	0	4.74	< 0.001		5.627	1.619 - 19.562
Unknown	567.7004		7.36	< 0.001	−	0.140	0.036 - 0.538
Unknown	1090.25		7.78	0.002	−	0.172	0.040 - 0.750
PE(36:1)	745.5606	8	7.86	0.001	−	0.186	0.048 - 0.726
Unknown	766.5294		8.70	0.006	−	3.937	1.076 - 14.404
Unknown	1674.449		9.75	0.003	−	5.029	1.216 - 20.796
Unknown	362.2429		3.16	< 0.001	−	6.488	1.840- 22.884
Unknown	337.3336		5.53	0.005	−	3.871	1.123 - 13.342
PS(40:4)	899.5815	0	7.08	0.005	−	0.277	0.086 - 0.893
Unknown	795.9053		7.36	< 0.001	−	0.090	0.022 - 0.336
Unknown	671.739		7.37	0.005	−	3.510	1.104 - 11.157
Unknown	586.492		3.84	< 0.001	−	12.73	2.450 - 66.191
Unknown	821.1918		6.05	< 0.001	−	0.043	0.006 - 0.312
Unknown	851.9697		7.35	< 0.001	−	0.060	0.011 - 0.347
PC(O-32:1)/PC(P-32:0)	777.5732	10	7.76	0.004	−	5.836	1.185 - 28.754
PC(O-34:1)/PC(P-34:0)	805.6179	7	7.89	0.001	−	11.30	1.802 - 70.868
Unknown	729.5663		8.12	< 0.001	−	0.022	0.003 - 0.186
Cer(d18:1/22:0)	621.6035	9	8.72	0.003	−	6.751	1.310 - 34.798
Unknown	1156.334		8.73	0.001	−	6.524	1.563 - 27.239
Unknown	1616.381		9.70	< 0.001	−	9.011	1.821 - 44.596
Unknown	382.372		6.99	0.010	−	4.465	0.993 - 20.070

RT: retention time (min); IM: ionization mode; OR: Odds ratio
Delta = (abs(query mass - adduct mass)/adduct mass)*1000000MS

© 2023. Dakterzada F et al. Published online at http://www.aginganddisease.org/EN/10.14336/AD.2023.0220

SUPPLEMENTARY DATA

Supplementary Table 3. Plasma lipids associated with the positivity of each AD-related CSF biomarker.

	Lipid name	Mass	Delta (ppm)	RT	IM	*p*	OR	99% CI for OR
Aβ42	**Unknown**	1493.132		7.40	+	0.001	4.498	1.403 – 14.424
	**Unknown**	1186.342		8.07	+	0.001	3.837	1.310 - 11.233
	**Unknown**	800.6758		8.66	+	< 0.001	0.184	0.057 – 0.599
	**Unknown**	1709.463		10.1	+	< 0.001	0.176	0.053 – 0.577
	**Unknown**	674.6725		10.68	+	< 0.001	4.892	1.591 – 15.041
	**TG(56:2)**	931.8369	27	10.07	+	0.005	0.350	0.134 – 0.915
	**PS(42:3)**	851.595	0	7.96	+	0.003	0.302	0.106 – 0.860
	**Unknown**	1482.414		8.88	+	< 0.001	4.604	1.682– 13.270
	**TG(55:1)**	922.6617	19	7.95	+	0.007	2.800	1.042 – 7.528
	**PC(P-42:4)/PC(O-42:5)**	849.6643	0	8.35	+	0.001	3.373	1.282 – 8.876
	**Unknown**	1719.455		10.53	+	< 0.001	4.152	1.478 – 11.669
	**PC(O-38:2)**	829.619	1	7.89	−	0.002	3.312	1.242 – 8.834
	**Unknown**	2192.572		10.24	−	0.001	3.592	1.304 – 9.894
	**PA(i-24:0/a-25:0)**	868.7177	3	10.27	−	0.003	0.313	0.115 – 0.848
	**TG(O-55:6)**	878.768	4	10.41	−	0.007	2.854	1.048 – 7.770
	**Unknown**	829.7762		8.26	−	0.004	3.071	1.132 – 8.331
	**Unknown**	1265.304		8.32	−	0.001	3.684	1.320 – 10.281
**Ptau**	**TG(O-64:7)**	984.8828	13	10.26	+	0.010	5.332	0.995 – 1.674
**Ttau**	**Unknown**	902.8486		7.31	+	0.009	2.712	1.017 – 7.230

Aβ42: amyloid beta 1-42; Ttau: total tau; Ptau: phosphorylated tau; RT: retention time (min); IM: ionization mode; OR: Odds ratio
Delta = (abs(query mass - adduct mass)/adduct mass)*1000000MS

Supplementary Table 4. The frequency of comorbidities between patients with pathological and nonpathological levels of Aβ42 in CSF.

	Total (N = 199)	Aβ42+ (N = 124)	Aβ42- (N = 75)	*p*
**Depression**	32.6% (65)	33.8% (42)	30.6% (23)	0.64
**Hypertension**	57.7% (115)	59.6% (74)	54.6% (41)	0.488
**Stroke**	4.0% (8)	4.8% (6)	2.6% (2)	0.45
**Diabetes Melitus**	20.6% (41)	17.7% (22)	25.3% (19)	0.199
**Dyslipidemia**	40.2% (80)	37.0% (46)	45.3% (34)	0.251

Aβ42: amyloid beta 1-42; Aβ42+: Aβ42≤600 pg/mL; Aβ42-: Aβ42>600 pg/mL; *P* values were calculated by comparing groups Pearson's Chi-square test for qualitative variables.

Supplementary Table 5. The frequency of comorbidities between patients with pathological and nonpathological levels of Ttau in CSF.

	Total (N = 198)	Ttau+ (N = 95)	Ttau- (N = 103)	*p*
**Depression**	32.8% (65)	31.5% (30)	33.9% (35)	0.719
**Hypertension**	57.5% (114)	53.6% (51)	61.1% (63)	0.287
**Stroke**	4.0% (8)	4.2% (4)	3.8% (4)	0.907
**Diabetes Melitus**	20.7% (41)	21.0% (20)	20.3% (21)	0.908
**Dyslipidemia**	39.8% (79)	41.0% (39)	38.8% (40)	0.75

Ttau: total tau; Ttau+: Ttau >425 pg/mL; Ttau-: Ttau ≤425 pg/mL; *P* values were calculated by comparing groups using Pearson's Chi-square test for qualitative variables.

Supplementary Table 6. The frequency of comorbidities between patients with pathological and nonpathological levels of Aβ42 in CSF.

© 2023. Dakterzada F et al. Published online at http://www.aginganddisease.org/EN/10.14336/AD.2023.0220

SUPPLEMENTARY DATA

	Total (N = 198)	Ptau+ (N = 106)	Ptau- (N = 92)	*p*
**Depression**	32.8% (65)	29.2% (31)	36.9% (34)	0.249
**Hypertension**	57.5% (114)	51.8% (55)	64.1% (59)	0.082
**Stroke**	4.0% (8)	3.7% (4)	4.3% (4)	0.838
**Diabetes Melitus**	20.7% (41)	19.8% (21)	21.7% (20)	0.738
**Dyslipidemia**	39.8% (79)	41.5% (44)	38.0% (35)	0.619

Ptau: phosphorylated tau; **Ptau+**: Ptau>65 pg/mL; Ptau-: Ptau≤65 pg/mL; *P* values were calculated by comparing groups Pearson's Chi-square test for qualitative variables.

Supplementary Table 7. Plasma lipids associated with progression from MCI to AD.

Lipid name	Mass	Delta (ppm)	RT	IM	*p*	OR	99% CI for OR
**11,12-EET**	380.2599		4.61	−	0.001	54.985	2.160 – 1399.431
**PS(40:4)**	899.5815	0	7.08	−	0.004	13.418	1.352 – 133.195
**Unknown**	651.7253		7.35	−	0.002	0.056	0.005 – 0.616
**TG(53:4)**	928.7665	1	10.1	−	0.005	0.030	0.001 – 0.730
**PC(44:10)**	927.6163	27	7.64	−	0.007	5.979	1.088 – 32.867
**Unknown**	1120.717		9.06	−	0.001	10.847	1.712 – 68.740

RT: retention time (min); IM: ionization mode; OR: Odds ratio
Delta = (abs(query mass - adduct mass)/adduct mass)*1000000MS

Supplementary Table 8. Plasma lipids associated with rate of progression from MCI to AD.

Name	Mass	Delta (ppm)	RT	IM	*p*	OR	99% CI for OR
**APOE ε4**					< 0.001	12.383	4.137 – 37.065
**Unknown**	2294.667		7.03	+	0.003	0.342	0.135 – 0.870
**PC(44:5)**	891.6419	13	7.38	+	< 0.001	9.884	3.081 – 31.706
**TG(60:6)**	1045.868	17	9.99	+	< 0.001	0.164	0.056 – 0.479
**TG(48:1)**	821.7482	0	10.07	+	< 0.001	0.153	0.056 – 0.419
**Unknown**	1734.557		10.26	+	< 0.001	34.806	4.879 – 248.30
**Unknown**	1760.574		10.27	+	0.001	0.148	0.034 – 0.644
**TG(61:7)**	973.8682	0	10.26	+	< 0.001	0.029	0.007 – 0.131
**Aβ42**					< 0.001	0.996	0.994 – 0.998
**Ttau**					< 0.001	1.005	1.003 – 1.008
**Unknown**	302.26		4.62	−	< 0.001	5.766	2.069 – 16.06
**PS(40:4)**	899.5815	0	7.08	−	< 0.001	14.214	4.166 – 48.50
**PC(36:2)**	831.5991	9	7.32	−	0.003	0.287	0.097 – 0.853
**Unknown**	446.0925		7.60	−	< 0.001	0.239	0.087 – 0.655
**Unknown**	662.7532		7.84	−	0.003	0.295	0.101 – 0.862
**Unknown**	1460.338		8.88	−	< 0.001	0.097	0.031 – 0.309
**Unknown**	1038.717		9.06	−	< 0.001	3.668	1.419 – 9.477

RT: retention time (min); IM: ionization mode; OR: Odds ratio
Delta = (abs(query mass - adduct mass)/adduct mass)*1000000MS

© 2023. Dakterzada F et al. Published online at http://www.aginganddisease.org/EN/10.14336/AD.2023.0220
